# Circular Suture Ligation of Presacral Venous Plexus to Control Presacral Venous Bleeding During Rectal Mobilization

**DOI:** 10.1007/s11605-012-2028-x

**Published:** 2012-09-21

**Authors:** Jinbo Jiang, Xuemei Li, Yanlei Wang, Hui Qu, Zutao Jin, Yong Dai

**Affiliations:** 1 Department of General Surgery, Shandong University Qilu Hospital, Jinan, Shandong China; 2 School of Medicine, Shandong University, Shandong, China

**Keywords:** Circular suture ligation, Presacral bleeding, Rectal mobilization, Rectal cancer

## Abstract

**Background:**

Presacral venous bleeding during rectal mobilization is uncommon but potentially life-threatening. Various methods have been proposed for controlling the bleeding, but each has some obvious limitations in clinical practice. We report a simple technique that was designated as circular suture ligation. This technique was efficient in controlling presacral venous bleeding encountered during rectal mobilization.

**Methods:**

The key point of circular suture ligation was to control the bleeding by suture ligating the venous plexus in one or more circles in the area with intact presacral fascia that surrounds the bleeding site while the bleeding site was temporarily controlled with fingertip pressure. From September 2007 to December 2011, 258 patients underwent rectal surgery in our department because of rectal cancer. Uncontrolled presacral venous bleeding with traditional methods was encountered in eight patients (3 %) with estimated blood loss from 300 to 5,000 ml.

**Results:**

Bleeding was successfully controlled in all eight patients with the circular suture ligation. None of the patients required reoperation for bleeding or other issues. No patients developed chronic pelvic pain after the operation.

**Conclusions:**

Our experience suggests that circular suture ligation of venous plexus in the area with intact presacral fascia that surrounds the bleeding site is an effective and simple technique to control presacral venous bleeding when traditional techniques fail.

## Introduction

**Fig. 1:**
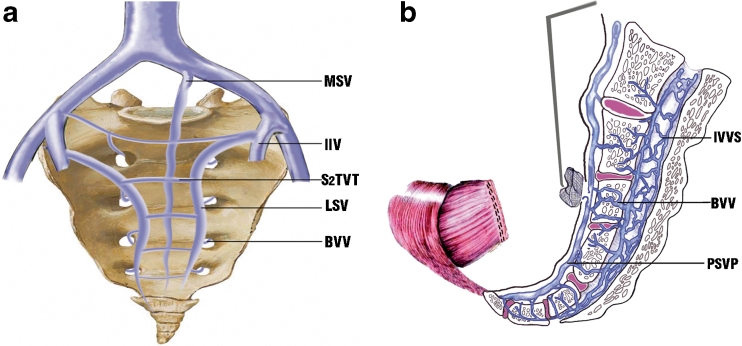
Diagram of the (**a**) presacral venous plexus and the (**b**) bleeding site pressed with small gauze along with rapid resection of the rectum. *MSV* middle sacral vein, *IIV* internal iliac vein, *S*_*2*_ *TVT* S_2_ transverse venous trunk, *LSV* lateral sacral vein, *BVV* basivertebral vein, *IVVS* internal vertebral venous system, *PSVP* presacral venous plexus

Presacral venous plexus lies posterior to the fascia propria of the rectum and underneath the presacral fascia, presenting in a ladder-like fashion (Fig. 1).^1^–^3^ Because of these anatomical features, the presacral veins are easily lacerated when the presacral fascia is lifted or disrupted during pelvic dissection and tend to bleed massively.^4^,^5^ Unfortunately, the presacral venous bleeding is often difficult to be controlled and can be life-threatening, although it is uncommon (incidence, 3–9 %).^1^

Several methods are commonly used for controlling bleeding such as pelvic packing and sterile thumbtacks. Pelvic packing with laparotomy pads requires removal of the packs later, which increases the risk of recurrent hemorrhage, anastomotic disruption, and pelvic infection.^6^,^7^ Sterile thumbtacks require the availability of special equipment and are often ineffective for diffuse hemorrhage.^8^,^9^ Several alternative methods have also been proposed such as the use of bone wax and the coagulation or co-suturing of bleeding veins with muscle fragments. Each method has certain limitations. Herein, we report the use of a simple and effective technique, designated as circular suture ligation, for controlling presacral venous bleeding during rectal mobilization. The key component of this technique was to control the bleeding by suture ligating the venous plexus in one or more circles in the area with the intact presacral fascia that surrounds the bleeding site(s).

## Materials and Methods

### Patients

A total of 258 patients underwent open rectal resection because of rectal cancer from September 2007 to December 2011 in our hospital. Of them, 189 patients had a low anterior resection, and 69 patients had an abdominoperineal resection. Eight patients (3 %) had uncontrolled presacral venous bleeding (Table 1): five were males, three were females; the mean age of the eight patients was 56 years old (range 38–75 years old). Low anterior resection was the primary procedure for four patients, and abdominoperineal resection was for the other four patients.

**Table 1:** Information on the patients with presacral bleeding during rectal mobilization

Patient	Sex	Age (years)	TNM stage	Surgical procedure	Blood loss (mL)	Blood transfused (unit)	Postoperative complication
1	M	52	T3N2M0	LAR	1,300	8	Thrombosis in LLE
2	F	38	T2N1M0	APR	600	2	None
3	F	66	T3N0M0	LAR	300	0	None
4	M	63	T3N0M0	APR	300	0	Blood loss 650 ml
5	M	75	T3N2M0	APR	5,000	24	None
6	M	54	T3N0M0	LAR	300	2	None
7	F	44	T3N2M0	APR	1,000	4	None
8	M	56	T3N1M0	LAR	400	0	None

*APR* abdominoperineal resection, *F* female, *LAR* low anterior resection, *LLE* left lower extremity, *M* male, *TNM* tumor node metastasis

### Surgical Technique of Circular Suture Ligation

Circular suture ligation was performed in all eight patients in order to control the presacral venous bleeding. The procedure of the circular suture ligation could be divided into three continuous steps.

First, immediate direct pressure was applied over the bleeding site using fingertip or small tampon gauze to temporarily control the bleeding (Fig. 1b). The bleeding site was kept as dry as possible by maintaining an effective suction. At the same time, the anesthesiologist and nurses were notified on the extent of bleeding and the necessity to prepare blood for further hemorrhage.

Second, the rectum with the tumor was removed quickly while controlling the bleeding with pressure, thus the bleeding site could be exposed thoroughly. If the sphincter-preserving procedure was feasible, the rectum was then dissected rapidly and transected with a cutter stapler at the site of the estimated tangent distal to the tumor. Otherwise, abdominoperineal resection of the rectum was performed. After the removal of the rectum, the details of the presacral fascia injury, especially the denuded veins, could be observed in five cases. In the other three cases, the blood vessels were less clear or could not be observed.

**Fig. 2:**
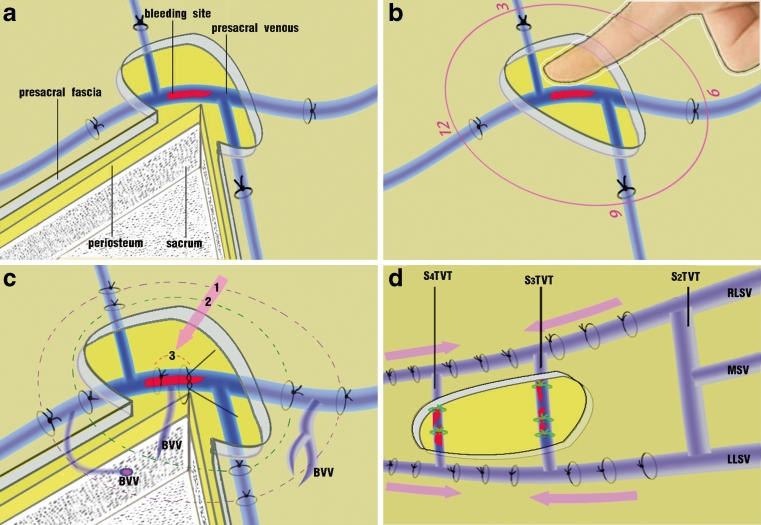
Demonstration of the procedure of circular suture ligation for bleeding control. **a** The bleeding site (*red color*). **b** Direct pressure on the bleeding spot using fingertip, and then one round of circular suture ligation at 90- or small-degree intervals was performed in the area with intact presacral fascia around the fingertip. The suture-ligated tissues should include the presacral fascia, the presacral veins, and the deep connective tissues. **c** Multiple rounds of circular suture ligation to control bleeding. The numbers *1*, *2*, and *3* represent the first, second, and third rounds of circular suture ligation, respectively. The *pink arrow* indicates the direction of the circular suture ligation from a distance to the bleeding site. **d** The ligation of both lateral sacral veins in the area with intact presacral fascia in patient 5. The *pink arrows* indicate the direction of the suture ligation. *BVV* basivertebral vein, *RLSV* right lateral sacral vein, *LLSV* left lateral sacral vein, *MSV* middle sacral vein, *S*_*2–4*_ *TVT* S_2–4_ transverse venous trunk

Third, suture ligation of the presacral veins in circles was performed around the bleeding site while continuous pressure over the bleeding site was given using the fingertip (Fig. 2a, b). In the case that the venous branches surrounding the fingertip could be identified, they were suture ligated one by one with a narrow, tapered needle and 4–0 silk suture thread. Importantly, the suture-ligated tissues should include the presacral fascia, the presacral veins, and the deep connective tissues. Suture ligation should first be performed in the area where the presacral fascia was intact. After the first circular suture ligation, the fingertip was moved away carefully to observe whether the bleeding was under control. If the bleeding was largely controlled, then the bleeding spot was further pressed for a while with hemostatic gauze (Johnson & Johnson, NJ), and the bleeding usually stopped.

Continuous bleeding after the first circular suture ligation often suggested that the bleeding was from the deep communicating veins or from a retracted vein inside the bone. In this situation, the second circular suture ligation about 0.5 cm inside the first circular suture was performed, and if necessary, the third circular suture ligation was then given until the bleeding stopped (Fig. 2c). In the case that the presacral veins were hardly identified, suture ligation was performed at 90- or smaller-degree intervals around the fingertip. The presacral veins are typically distributed with a ladder-like pattern, which could help determine where to suture.

## Results

In the present group of patients, 13 patients (13/258) had presacral venous bleeding. In five patients (5/258), the bleeding stopped with temporary packing using hemostatic gauze. In eight patients (8/258), the bleeding control failed with the use of pressure. The estimated blood loss in the eight patients ranged from 300 to 5,000 ml, and five of them received blood transfusion with 2–24 U of blood (Table 1).

In seven of the eight patients, one bleeding site was observed, and the bleeding was successfully controlled in six patients with circular suture ligation (Table 1). For the remaining one patient (no. 1 in Table 1), the blood vessel was torn during suturing, which resulted in a new bleeding site. The two bleeding sites were then controlled separately with circular suture ligation. The estimated blood loss in the seven patients ranged from 300 to 1,300 ml, and blood (2–8 U) was transfused to four of them. After operation, one patient had 600 ml more blood loss from the presacral drainage just in several minutes after the negative pressure drainage bag was applied. But the bleeding stopped after the negative pressure drainage was replaced with urine bag. Other patients had only minimal blood loss.

One of the eight patients (no. 5 in Table 1) had a large area laceration of the presacral fascia, which led to massive bleeding of the S_3_ and S_4_ transverse veins. The initial circular suture ligation in the lacerated presacral fascia resulted in further vessel laceration and hemorrhage. Metallic thumbtacks were then applied but failed to effectively control the bleeding. Failure with metallic thumbtacks might be partially due to the relatively large area of the laceration and diffuse hemorrhage. We packed the bleeding area with small gauze, suture ligated both the lateral sacral veins outside the area of defect presacral fascia, and the bleeding was controlled (Fig. 2d). The blood loss was estimated to be 5,000 ml, and 24 U of blood was transfused to this patient. This patient had an uneventful course after the operation.

Postoperatively, all patients were followed closely and the mean follow-up time was 20 months (8–38 months). None of the eight patients required reoperation for bleeding, and none had chronic pelvic pain. During hospitalization, one patient (no. 1 in Table 1) developed deep venous thrombosis in the left lower extremity. We believe that the thrombosis was not caused by the circular suture ligation, but occurred as an unrelated complication. We do not regularly use low molecular weight heparin to prevent deep venous thrombosis before operation.

## Discussion

The presacral venous bleeding during rectal mobilization is rare, but such bleeding can be massive and fatal. The pelvic gauze packing is effective and remains the first choice for lifesaving in catastrophic situations. However, there are some obvious disadvantages for pelvic packing such as the need to remove the packs later, increased risks of infection and anastomotic disruption, and delay of wound healing.^6^,^7^,^10^ Sterile metallic or titanium thumbtacks are also effective for stopping bleeding. However, the use of thumbtacks necessitates permanent implantation of a foreign body into the body with the potential of thumbtack displacement. The application of thumbtacks is often difficult at the levels of S_3_ and S_4_ because of the anatomical curvature of the sacrum. Failure to control bleeding with thumbtacks in one patient of our group was an example for its limitations. Furthermore, thumbtacks are not readily available in all hospitals.^4^,^8^ Several other methods have also been described in the literature, and each has some limitations.^11^–^14^

The circular suture ligation method we developed had two key points in performance. First, the suture ligation of the presacral veins should be first placed in the area with intact presacral fascia surrounding the bleeding site. Because the presacral venous wall is too thin to bear the cut and pull from suture ligation procedure, each presacral vein must be suture ligated together with the superficial presacral fascia and the deep connective tissues. The presacral fascia and deep connective tissues could effectively protect the thin-walled presacral veins from being torn. Second, suture ligation was performed in a circle around the bleeding site, and the next circular suture ligation could be carried out inside the previous circle of the suture if bleeding continues. After the first circular suture ligation, the bleeding in most cases was largely controlled. If bleeding continues, it suggests that the bleeding come from the deep sacral rami communicans inside the first circle. In this situation, the second circular suture ligation inside the first circle could probably occlude these rami and reduce the bleeding significantly, which even makes the direct suture ligation of the bleeding vessels possible.

After the rectum is removed, the blood vessels are often faintly visible through the presacral fascia. In the case that the blood vessels are hardly identified, the typical ladder-like anatomical feature can serve for the judgment of vein locations, and suture ligation can be carried out at 90- or smaller-degree intervals around the fingertip. If blood vessels are torn due to mis-manipulation, another circle of suture ligation around the new bleeding site could be performed to stop the bleeding.

We noticed some potential limitations for the circular suture ligation technique. First, circular suture ligation can be difficult for bleeding occurring at the bottom of a narrow pelvis, typical in patients with obesity. Rapid removal of the rectum and maintenance of efficient suction will help in identifying the bleeding site and in performing circular suture ligation. Preoperative radiotherapy was not performed in our group. Long-course preoperative radiotherapy may potentially lead to the delay of wound healing and difficulties of surgical dissection and suture ligation. Second, individual differences in vessel distribution may become an issue when the vessels are difficult to be identified. The vein locations judged based on the typical pattern of vein distribution could be wrong, leading to failure of bleeding control. However, in our experience, another circle of suture ligation is often effective. Third, for bleeding coming from retracted vein inside the bone, we think that the combined use of suture ligation, packing with hemostatic gauze, and other techniques is more efficient than single method to control the bleeding in this situation.

In summary, we applied the technique of circular suture ligation to control presacral venous bleeding during rectal mobilization, and bleeding was successfully arrested in all eight patients. None of the patients required reoperation for secondary bleeding or other issues. No patient developed chronic pelvic pain after operation (follow-up time from 8–38 months), suggesting that the circular suture ligation did not cause severe injury to vital structures such as nerves. Our experience indicates that the circular suture ligation is simple, effective, and cheap for controlling presacral venous bleeding when traditional methods fail.
